# Exploring Actual and Presumed Links between Accurately Inferring Contents of Other People’s Minds and Prosocial Outcomes

**DOI:** 10.3390/jintelligence12020013

**Published:** 2024-01-26

**Authors:** Sara D. Hodges, Murat Kezer, Judith A. Hall, Jacquie D. Vorauer

**Affiliations:** 1 Department of Psychology, University of Oregon, 1451 Onyx Street, Eugene, OR 97403-1227, USA; mkezer@uoregon.edu; 2 Department of Psychology, Northeastern University, Boston, MA 02115, USA; j.hall@northeastern.edu; 3 Department of Psychology, University of Manitoba, Winnipeg, MB R3T 2N2, Canada; jacquie.vorauer@umanitoba.ca

**Keywords:** interpersonal sensitivity, empathic accuracy, mentalizing, prosocial behavior

## Abstract

The term “empathic accuracy” has been applied to people’s ability to infer the contents of other people’s minds—that is, other people’s varying feelings and/or thoughts over the course of a social interaction. However, despite the ease of intuitively linking this skill to competence in helping professions such as counseling, the “empathic” prefix in its name may have contributed to overestimating its association with prosocial traits and behaviors. Accuracy in reading others’ thoughts and feelings, like many other skills, can be used toward prosocial—but also malevolent or morally neutral—ends. Prosocial intentions can direct attention towards other people’s thoughts and feelings, which may, in turn, increase accuracy in inferring those thoughts and feelings, but attention to others’ thoughts and feelings does not necessarily heighten prosocial intentions, let alone outcomes.

## 1. Introduction

The term “empathic accuracy” has been applied to the accurate inference of the dynamically changing contents of other people’s minds or the “subjectively perceived mental events of another person as they occur over time” (Hodges et al. 2015, p. 319). As research on this topic has grown, the empathic prefix on this term has proven problematic. It suggests that accurately knowing others’ thoughts and feelings is part of having their best interests at heart, caring for them, or, more generally, being “good”. However, the name seems to carry more prosocial promise than this form of interpersonal accuracy delivers. Furthermore, the empathic label appears to have guided assumptions about what “should” be correlated with this form of accuracy and has driven the contexts in which it has been studied. Researchers of this form of accuracy may be a little guilty of looking under the lamppost—disproportionately studying accuracy at inferring other people’s thoughts and feelings in participants who would have reason to care for and feel compassion for the people whose thoughts and feelings they are inferring. However, to foreshadow, we also think there may be some bias in positioning the lamppost to highlight and feature certain results and obscure others.

At first blush, the prosocial nature of the term empathic accuracy seems intuitive and fitting: Other people who can accurately read our minds and infer our thoughts and feelings would seem to have access to our inner selves—access that allows them to understand us in a way that resembles their understanding of their own selves. Indeed, previous research on “empathic accuracy” involves one person recognizing and contemplating another person’s perspective, feelings, and experience with at least some of the attention and *possibly* the favor that the first person habitually applies to themselves. However, the empathic prefix in “empathic accuracy” primes assumptions of caring and benevolent motivations and intentions destined for prosocial outcomes along with that accuracy. Recognition and contemplation of another person’s thoughts and feelings, even if they yield accurate inferences, do not always assure compassionate understanding or action to help that other person. To emphasize this point and to establish a more neutral starting point from which to explore the relationship between accuracy in inferring others’ thoughts and feelings and prosocial outcomes, we will use the term “thought-feeling accuracy” throughout this paper, unless we are intentionally referring to the historical trajectory this construct passed through as “empathic accuracy”. 

We will also limit our focus to research using a paradigm initially billed as capturing “empathic accuracy” that measures dynamic thought-feeling accuracy in ongoing social communication. In this paradigm, perceivers’ inferences of a target person’s thoughts and feelings are scored for accuracy using the target person’s own reports as the criterion. Thus, this thought-feeling accuracy is an ability-related state, not a personality trait. As an ability measured within interpersonal interactions, thought-feeling accuracy involves, among other things, being able to perceive, access, generate, and understand emotions, all components of emotional intelligence, as the construct was initially outlined by Mayer and Salovey (1997). Notably, the term “empathic accuracy” has been applied beyond its original, intended meaning to refer to other ability-related measures of emotional intelligence, specifically standardized tests measuring the ability to decode emotion expressions and nonverbal cues, particularly ones using items that require the test-taker to identify posed emotion expressions. The overlapping elements of thought-feeling accuracy and accuracy at decoding nonverbal and affective cues have earned both forms of accuracy a place in the discussion of “emotional intelligence”. Furthermore, and of relevance for this Special Issue (see Mortillaro and Schlegel 2023), both forms of accuracy are measures of ability rather than personality traits. Emotion decoding accuracy has also been linked—although not consistently in empirical results—to prosocial behavior and (often self-reported) measures of empathy-related constructs (see Hodges and Wise 2016; Mayukha et al. 2020; Olderbak and Wilhelm 2017; Schlegel 2020). 

However, thought-feeling accuracy and emotion decoding accuracy also differ; among other things, the latter is generally measured using static stimuli (e.g., a photo of a facial expression), often made from actors posing emotional expressions outside of natural social interactions (e.g., Gur et al. 2002; Nowicki and Duke 1994; Schlegel et al. 2014) or posing for other purposes (e.g., for advertisements, such as the Reading the Mind in Eyes Task (RMET)^1^, (Baron-Cohen et al. 2001); but there are some exceptions, e.g., (Costanzo and Archer 1989)). Additional coverage of some measures of emotion recognition and nonverbal decoding, along with their applications to emotional intelligence, appears elsewhere (e.g., Buck et al. 2017) and in this issue (e.g., Mortillaro and Schlegel 2023).

In what follows, we first provide some background about measuring thought-feeling accuracy in social interactions. We then examine why a clever and enduring paradigm for measuring thought-feeling accuracy both set the table and tilted it towards assumptions of “empathic” accuracy, which helped create and perpetuate persistent assumptions that have affected the direction of research and conclusions that followed. Next, we consider potential realms of thought-feeling accuracy that are unrelated to prosocial behavior—or even related to harmful behaviors—and finally, we consider directions for future study. 

## 2. Measuring Thought-Feeling Accuracy

William Ickes, the researcher who initially popularized the term empathic accuracy, has also referred to it as everyday mindreading (e.g., Ickes 2003). Although talking about mindreading runs the danger of evoking a woo-woo element of psychic abilities, it does capture key defining elements of the specific type of interpersonal accuracy we address here: a perceiver inferring a target person’s dynamic thoughts and feelings as they unfold over time in the context of a social interaction. Measures of thought-feeling accuracy share similarities with other measures of interpersonal accuracy, such as accuracy in assessing people’s personality traits (e.g., Letzring 2008). However, unlike thoughts and feelings, personality traits change less, and when they do, they change relatively slowly. In addition, the criterion for accuracy in assessing another person’s personality traits may come from the target’s self-report, informant reports, or other personality measures (including projective or physiological measures). In contrast, due to the subjective nature of thoughts and feelings, the criterion for thought-feeling accuracy, more or less by necessity, has to be provided by the target person who has them. 

The original “empathic accuracy” paradigm, as developed by Ickes and colleagues (e.g., Ickes et al. 1990), entails video-recording a *target* person who is taking part in a conversation or responding to questions. The target then watches the video recording, which is stopped at specific points (either those selected by the target or at arbitrary time intervals), and the target is asked to report what they were thinking and feeling at each stop point. The video recording is then shown to *perceivers*, the people whose accuracy is being measured, and it is stopped at the same stop points, where perceivers are asked to provide their best guess of what the target was thinking or feeling. Independent coders then compare the target’s reported thoughts and feelings to the perceiver’s inferences and rate them for accuracy using a 3- or 4-point scale (Ickes et al. 1990; Hodges et al. 2015). The Ickes paradigm is further divided into two sub-paradigms: the *Standard Stimulus* sub-paradigm, in which perceivers who were not part of the original interaction see stimuli that were recorded earlier, and the *Dyadic Interaction* sub-paradigm, in which the perceiver is one of the interactants in the recorded interaction. In the Dyadic Interaction sub-paradigm, participants may be both targets and perceivers—both recording their own thoughts and feelings and also inferring those of the other person in the dyad. This method also highlights how individual thoughts and feelings (not just perceivers) may be used as an interesting unit of analysis. 

Another paradigm introduced by Levenson and Ruef (1992) yields what has been called “empathic accuracy” but is considerably narrower, focusing on accuracy at perceiving changes in the valence of the target’s affect (not thoughts). It has been used more recently by Zaki and colleagues (e.g., Zaki et al. 2008) and adapted in various ways by others (e.g., to capture changes in specific emotions like anger or happiness—see Eckland et al. 2020; McKenzie et al. 2022). It also involves a video recording, usually of a target describing an emotional experience. The target then views this video recording and is given a continuously adjustable slider or dial on which to rate the valence of their affect on a multi-point (e.g., 9-point) scale from extremely negative to extremely positive. Perceivers watch the same videos and also continuously rate what they believe is the valence of the target’s affect. Accuracy is either the correlation or difference scores between the target’s self-ratings of affect valence and the perceiver’s inferred ratings of the target’s affect across time. To use terminology borrowed from the other (Ickes) paradigm, the Levenson–Ruef–Zaki paradigm generally uses “Standard Stimuli” (i.e., videos that are a few minutes long), although the method could be used with a video of an interacting dyad (e.g., by instructing perceivers to focus on guessing the valence of affect for the other person in the dyad, as in Lewis 2014). 

Thus, both the Ickes and the Levenson–Ruef–Zaki paradigms capture elements of the subjective and dynamic experience of what is going through another person’s mind, and they both track these changing mental states in concert with what the target is outwardly sharing. However, the Ickes and the Levenson–Ruef–Zaki paradigms also differ from each other. Interestingly, in one study, we know that when a researcher measured both and looked at the correlation between the two, they were uncorrelated (Lewis 2014). The Levenson–Ruef–Zaki method tracks changes in what the target is experiencing but prioritizes accuracy in seeing changes in the valence of affect. In contrast, the Ickes method arguably more closely resembles what people naturally find themselves thinking about when wondering what others think. The Ickes method measures perceivers’ ability to accurately capture something akin to the director’s commentary in a movie—except in this case, that commentary is about the “life-movie” that runs through a target’s head. The Ickes method gives perceivers credit for being accurate at knowing what the target is thinking about and which emotion the target is feeling, not just for knowing how positive or negative the target is feeling. Because the Ickes method tackles this more detailed form of “mindreading”, we concentrate primarily on the Ickes method throughout the rest of this paper. 

Lay assumptions about accuracy in an ongoing social interaction seem to rely a lot on nonverbal decoding (i.e., reading body language). However, thought-feeling accuracy has been demonstrated in past studies to rely more heavily on the target’s verbal responses than nonverbal ones (Gesn and Ickes 1999; Hall and Schmid Mast 2007; Hodges and Kezer 2021). To be clear, we are not dismissing the value of being able to accurately decode nonverbal emotion cues; only that this ability is not synonymous with thought-feeling accuracy, which draws on additional other cues as well. Notably, in the few studies we know of that measure both, accuracy for reading other people’s dynamic thoughts and feelings has been found to be unrelated to common tests of nonverbal decoding (e.g., Flykt et al. 2021). 

## 3. The Central Question

When Ickes developed this popular method for measuring thought-feeling accuracy, there was nothing in the methodology that limited accuracy to serving prosocial ends: accuracy was accuracy, not “empathic” accuracy. Indeed, one of the very first studies using the paradigm (Ickes et al. 1990) demonstrated that people are more accurate at inferring the thoughts and feelings of people they found attractive (and were interested in), something that would be hard to characterize as prototypically prosocial and is more easily viewed as self-serving (e.g., “I want to know what’s going on in that pretty little head of yours”). So why was this skill labeled empathic accuracy and not, for example, exploitative accuracy? We will provide six answers to that question in the second half of this paper. First, though, consistent with our central thesis that thought-feeling accuracy far from guarantees prosociality, we argue that what separates empathic accuracy and exploitative accuracy (which are likely two poles along a continuum) is *why* the perceiver tries to infer the target’s thoughts and feelings.

## 4. Motivation and Room for Improvement 

Motivation would appear to play a key role in the relationship between thought-feeling accuracy and prosociality. The question of whether greater motivation leads to greater accuracy has yielded mixed results, showing both improvement and impediment attributed to motivation (e.g., see Berlamont et al. 2023; Ickes et al. 1990; Klein and Hodges 2001; Lawless DesJardins and Hodges 2015; Simpson et al. 1995; Thomas and Maio 2008). Rather than looking for a linear relationship between motivation and accuracy, where each unit increase in motivation produces a corresponding increase in accuracy, it may instead be more fruitful to think about the critical level of motivation needed to trigger attempts to infer others’ thoughts and feelings. 

However, the important motivation question in relation to prosocial outcomes may be less “How motivated is the perceiver?” and more the question of “Motivated to do what?” In addition to the motivation simply to be accurate, the perceiver may also be motivated to accomplish other goals or support a particular belief. As is the case with many aspects of social perception, people are more likely to see (or, in this case, infer) what they want. For example, Simpson et al. (1995) found that people whose relationships were threatened by discussing how physically attractive other people were to their partners were more inaccurate at reading their romantic partners’ thoughts and feelings. We do not know from Simpson et al.’s results that participants’ inaccuracies were specifically of the sort that involved inferring that their partners were less attracted to these other people than the partners actually were, but the results would be consistent with this.

As simplistic as it sounds, we think that it is these different motives that figure largely in a model of when thought-feeling accuracy is related to prosocial or “empathic” behavior. As we will argue later, research exploring this “motivated to do what?” question has been limited because of the bias to look primarily for examples of “empathic” accuracy. An analogy to general intelligence seems apt: There is nothing inherently prosocial about intelligence, and the same seems to be true for accurately inferring others’ thoughts and feelings. Some people apply their gifts of intellect towards prosocial aims (e.g., coming up with ways to distribute healthcare resources to those who need them); others apply them to destructive and hurtful goals (e.g., figuring out ways to take other people’s money); a lot of people probably do at least a little bit of both. Similarly, the accurate apprehension of others’ thoughts and feelings can be put toward benevolent or malevolent goals, and the person who always infers thoughts and feelings for one reason or the other is likely rare. 

However, there are two caveats about how this analogy with general intelligence potentially breaks down. First, thought-feeling accuracy may be less “impartial” than the broad trait of general intelligence. Thought-feeling accuracy—because it is a specialized skill uniquely suited for interpersonal interactions and coordination—might predispose prosocial outcomes because it is deployed in situations that are critical for securing social belonging, something critical for our well-being (e.g., Baumeister and Leary 1995). Thought-feeling accuracy might be a bit like having accurate knowledge of what makes plants grow—this knowledge could be used for evil, but because the preponderance of intentional plant growth outcomes are good for humans, a green thumb is considered “good”. 

Second, there is the question of whether thought-feeling accuracy shows stable individual differences like intelligence does. There is less evidence for this than there is for the broader skill of emotion decoding (often a key component of emotional intelligence). At least two studies have shown some amount of variance in people’s thought-feeling inference scores that is attributable to perceivers across different targets (Lewis et al. 2012; Marangoni et al. 1995), which would be consistent with there being individual differences in thought-feeling accuracy. However, although these studies used multiple targets, there were some similarities in the targets’ situations. Thus, the variance that has been attributed to perceivers’ ability might be more attributable to the perceivers’ knowledge about or interest in a certain kind of target. Perhaps more importantly, few reliable correlates of thought-feeling accuracy have been found using the Ickes paradigm (see Hodges et al. 2015). In contrast, a variety of desirable traits have been associated with the more general skill of accurately perceiving emotions (see Hodges and Wise 2016, for a review). Furthermore, unlike nonverbal emotion decoding, where there is evidence that people can be trained to get better (e.g., Blanch-Hartigan et al. 2012; Schlegel et al. 2017), few interventions (with the possible exception of providing feedback—e.g., Barone et al. 2005; Lobchuk et al. 2016; Lobchuk et al. 2018; Lorimer and Jowett 2010a; Marangoni et al. 1995) have been identified that make people more accurate at inferring dynamic thoughts and feelings. 

Not being able to solidly claim that thought-feeling accuracy is a stable individual difference also undermines the ability to claim that people who would theoretically be more accurate would also be more prosocial. However, it is possible that more attempts at deploying accuracy (across different target individuals, as an individual difference) may lead to more prosocial outcomes. Unlike reading people’s emotional displays or listening to their words, there is no outward correlate with the contents of other people’s minds. Thus, inferring a person’s thoughts and feelings involves the integration of a variety of cues (including the target’s emotion displays and words—see Gesn and Ickes 1999; Hall and Schmid Mast 2007) and a fair amount of construction based on what we know about people in general (including the self); other people who are like the target (e.g., in terms of group membership or specific experiences); and any personal knowledge about a particular target’s history or idiosyncrasies. 

Taken together, there is a situation-specificity of the types of information and cues that can be used to inform inferences—and even of the kinds of integrations that need to be made and how much work is involved. Speculatively, thought-feeling accuracy may be connected to the broader idea of emotional intelligence in that emotionally intelligent people may know when it may be useful to try to infer others’ thoughts and feelings and also know a variety of tools that can be used to do this. Accuracy in inferring other people’s thoughts and feelings may be analogous to memorizing phone numbers: some people are better than others at doing it, but the more important variable in whether it happens or not may be whether people are moved to attempt it in the first place. It may be better to think about thought-feeling accuracy less as an individual difference that emerges across contexts and more as a tool that gets deployed in specific instances when a particular person finds it useful. Just as it would be cognitively taxing in ways that would prevent us from doing other things if we were to memorize every phone number we encountered, it similarly would not seem adaptive to infer the thoughts and feelings of every person we encounter. Fortunately, just as we do not *need* to memorize every phone number we encounter, there are a lot of things that go through other people’s minds that we do not need to infer—for any reason, let alone reasons aimed at being prosocial. 

## 5. Acquiring an (Undeserved?) Prosocial Glow 

What has led psychology researchers and laypeople alike to gravitate towards the view that people will use their “mindreading” skills for good? The belief that interpersonal sensitivity (including thought-feeling accuracy) is related to prosociality is pervasive and robustly recurrent—something the current group of authors have all encountered and have tried in various papers (sometimes collectively) to question. A chapter reviewing the relationship between prosocial behavior and a wide variety of ways to be interpersonally accurate (e.g., accuracy at emotion decoding; accurately identifying people’s traits, such as personality traits; and accuracy for other details such as what a person was wearing) suggested a much narrower and less robust relationship than commonly assumed (Hodges and Wise 2016). Similarly, a simple relationship between prosociality and perspective taking, the latter of which is frequently associated with both thought-feeling accuracy *and* the broad and prosocial concept of “empathy”, has also been questioned (see Sassenrath et al. 2022). Finally, specifically within the literature on thought-feeling accuracy that uses the Ickes paradigm, the idea that such accuracy can be used for less than prosocial goals has been raised but relatively neglected—perhaps because of the “empathic” label in the commonly used name for this paradigm (see Hall et al. 2021). So, why does the belief that thought-feeling accuracy is predictive of prosocial behavior seem to be stronger than the existing research evidence for such a link? We speculate about six reasons.

### 5.1. Reason 1: Thought-Feeling Accuracy Implies a Focus on Other People

The first reason is simple: attempts at thought-feeling accuracy share features with being “empathic”, a broad umbrella term used to describe separable and, quite frankly, often orthogonal components that are nonetheless intuitively associated (see Davis 1983; Hodges and Myers 2007; Zaki and Ochsner 2012). Interrupting our habitual self-focus to focus on another person’s thoughts and feelings may interrupt more self-centered and self-serving scheming. When this focus on another person lands on someone in need or distress, if we are accurate at inferring the other person’s thoughts and feelings (and the person has not otherwise told us what is wrong and why), those inferences will facilitate (though by no means guarantee) our ability to care for or help the other person. Moreover, given clear connections between perceived or expressed understanding and prosocial outcomes such as liking and feeling liked (e.g., Goldstein et al. 2014; Livingstone et al. 2020; Murray et al. 2002), it may seem natural to assume similar connections between *actual* understanding and such outcomes.

One of the key components under the empathic umbrella is perspective taking, and indeed, inferring another person’s thoughts and feelings at a specific moment does involve an attempt to apprehend part of their perspective, although perspective taking can also refer to attempts to capture someone else’s global opinions or general schemas. An act of perspective taking (or at least perspective consideration) is essentially built right into the process of inferring another person’s thoughts or feelings. Importantly, perspective taking plays a role in other constructs under the “empathic” umbrella. Manipulating perspective taking—or perhaps, as McAuliffe et al.’s (2020) meta-analysis suggests, manipulating the suppression of perspective taking (see also McAuliffe et al. 2018)—is thought to affect other constructs imbued with prosocial themes, particularly the “empathic concern” (also called sympathy and compassion) felt for another person and the helping and even altruistic behaviors that stem from feeling empathic concern (e.g., Batson et al. 2002; Batson et al. 2007). This is especially true when taking the perspective of targets who are objectively experiencing hardship (Coke et al. 1978). However, as we will see, attending to and accurately inferring another person’s thoughts and feelings does not always lead to compassion and helping, nor do effortful and conscious efforts to imagine another person’s point of view or imagine oneself in another’s place—see Sassenrath et al. 2022. 

### 5.2. Reason 2: There Are Instances When Thought-Feeling Accuracy and Prosociality Are Positively Correlated

We are not saying that there is *no* connection between thought-feeling accuracy and prosocial responding (“prosocial” is used here—and often elsewhere—broadly and inclusively, including everything from saying and doing nice things to incurring personal sacrifice to help others). However, the simple notion that people who can read other people well will also be helpful and kind to them may not be the best way to describe it. Before we elaborate more on what might be a better description, we want to acknowledge that we found a number of studies that could be seen as supportive of this association. 

Around the time we started writing this piece, we had reason (for another purpose) to compile a list of empirical articles that were indexed in PsycINFO, Medline/PubMed, and/or EBSCOhost and that both (1) mentioned empathic accuracy and (2) also used some variation of the Ickes paradigm (i.e., target reporting open response thoughts and feelings at specific time points; perceiver inferring those thoughts and feelings). While this list was not intended as a meta-analysis, nor was it exhaustive (for example, studies that used variants of the Ickes paradigm but did not label them as “empathic accuracy” were not included), it yielded an interesting set of 79 papers. We then went through the list, looking for studies that connected thought-feeling accuracy scores with prosocial outcomes—for which we used a very inclusive criterion. We came back with an assortment of 14 papers that documented a relationship between some variation of the Ickes paradigm and some outcome that could be seen as making things better for someone other than just the perceiver. (In some cases, this meant making things better for the target of accuracy; however, in other cases, prosocial outcomes were measured in different contexts or via trait measures.) Several of these prosocial outcomes were positive forms of support or responsiveness in romantic couples’ interactions (e.g., Hinnekens et al. 2018; Verhofstadt et al. 2016). Two papers used slightly more prototypical prosocial outcomes—e.g., coming up with a helpful accommodation for a health issue (Sened et al. 2020) or delivering effective counseling (Kwon and Jo 2012). Five papers (including two pairs of studies that each appeared to use the same samples) might be better described as measuring prosociality by looking for lower levels of antisocial behavior in the form of lower levels of reported aggression (e.g., Clements et al. 2007; Schweinle et al. 2002). 

Among the 14 papers with broadly prosocial outcomes, 13 showed either that higher levels of thought-feeling accuracy were correlated with more prosociality (e.g., Haas et al. 2015) or that lower levels of thought-feeling accuracy were related to aggression or being unsupportive. Only one of the 14 papers showed that a higher level of thought-feeling accuracy was related to a less prosocial outcome (specifically, more blaming—see Hinnekens et al. 2016). Among the 13 papers supporting the relationship, often there was some moderator or condition on the correlation (e.g., thought-feeling accuracy was related to higher scores on one subscale of an empathy measure but not other subscales—see Namba et al. 2021; or a result was present for male perceivers but not female ones—see Clements et al. 2007). 

Thus, in studies where both thought-feeling accuracy and some measure of prosociality (very broadly defined) are collected, the correlation is generally positive. However, that said, we think assuming that being attuned to others predicts prosocial behavior in a causal sense (an assumption some of us have frequently encountered when discussing our research: that a trait related to one definition of empathy is thought to bring about behavior related to another definition of empathy) probably does not do the best job of describing the association. Flipping the order may help a little^2^: valuing being (or just being) habitually helpful and nice as a broadly construed trait may lead one to have greater thought-feeling accuracy. Even then, empirical demonstrations of the relationship—causal or even just correlational—are often not straightforward (e.g., see Ickes et al. 1990; Issner et al. 2012; Namba et al. 2021; Verhofstadt et al. 2016; Zaki et al. 2008). A further possibility is that there are third variables, such as similarity or shared group membership between perceivers and targets, that independently help with thought-feeling accuracy and predict prosocial behavior towards the target. 

We think people try to maximize thought-feeling accuracy in moments when it matters to them. Prosocial people strive for thought-feeling accuracy when it affords them an opportunity to be prosocial because that is what they care about (see, for example, Winczewski et al. 2016; but consider also Izhaki-Costi and Schul 2011). But there is not an exclusive relationship between prosociality and thought-feeling accuracy: people high in relationship anxiety are more accurate in contexts that are threatening (indeed, see, for example, Simpson et al. 1999); people with a high need for achievement would be expected to be more accurate in contexts that might allow for achievement. Thus, it is perhaps not so surprising that trait measures of prosociality predict thought-feeling accuracy only inconsistently (as we have noted above). Individual differences on empathy and prosociality scales may do a better job of predicting thought-feeling accuracy in contexts where there are opportunities to be prosocial (e.g., listening to someone who has asked for advice or helping someone who is in distress)—and these instances would support an association between accuracy and prosocial behavior. Thus, there are other methodological refinements, such as collecting accuracy measures across different targets and different circumstances, that might better define the association.

### 5.3. Reason 3: Prosocial People Use Accuracy for Prosocial Ends

Our third reason why thought-feeling accuracy is assumed to correlate with prosocial behavior is a variation on the second reason. Just as prosocial people may engage in accuracy attempts in order to fulfill their prosocial goals, prosocial people have also probably learned techniques to increase accuracy that support their prosocial ways—just as they have learned to use other tools and strategies to support prosocial ends. For example, healthcare workers who are committed to healing others will have learned that listening carefully as another person describes symptoms leads to more effective healing attempts—a strategy that is consistent with the finding that perceivers whose attention is focused on a target’s words show better thought-feeling accuracy (attention to the verbal channel was manipulated in studies by (Gesn and Ickes 1999; Hall and Schmid Mast 2007); in a study by (Hodges and Kezer 2021), it was measured by examining how closely perceivers’ inferences matched what targets said out loud). Or a therapist may have learned over years of experience the benefits of drawing on generalizations about what people with a certain mental disorder think and feel (i.e., stereotypes—see also Lewis et al. 2012) to help treat a client who has that disorder. 

The important thing to remember is that people with other goals or orientations, even ones that may conflict with prosociality (e.g., self-serving goals), have probably also learned to be more accurate in support of those goals. For example, a salesperson trying to maximize her commissions may also carefully listen to a client or use generalizations about what members of certain groups tend to think when considering their product (e.g., “When people who work at the university buy a house, they always have concerns about which schools the house is zoned for…”) in order to increase the chances of closing a sale. There is nothing necessarily wrong or immoral about attending to or drawing on information that can make us more accurate in inferring the thoughts of others for self-serving purposes. However, it does highlight how there is nothing inherently proto-prosocial about being accurate. 

### 5.4. Reason 4: Thought-Feeling Accuracy Has Been Studied in Prosocially Relevant Contexts 

Our second and third reasons drew heavily on examples examining thought-feeling accuracy in contexts involving counseling or couples. Our fourth reason directly addresses this bias: research has disproportionately focused on specific contexts and relationships where accurate inference can clearly contribute to compassion and helpful behavior. Dubbing what has been studied extensively as “empathic accuracy” thus makes sense, given that much of the work has been done in settings where accuracy would likely be associated with caring and concern. Returning again to the list we compiled of 79 studies using the Ickes paradigm, we found that 30% (24 papers) measured thought-feeling accuracy in romantic couples (e.g., Berlamont et al. 2022; Crenshaw et al. 2019; Gadassi et al. 2011; Rafaeli et al. 2017; Sels et al. 2021). Another six papers measured it in perceivers who were caregivers or counselors (e.g., O’Brien and Haaga 2015; Reese et al. 2016), generally with targets who were experiencing some level of distress. Another line of research explored thought-feeling accuracy in perhaps the slightly less compassionate but still guidance-heavy relationship that occurs between coaches and athletes (e.g., Lorimer and Jowett 2009a, 2010b, 2011). 

In close relationships, significant others seem to read our minds to empathically deliver just what we need. This might mean picking out the perfect movie or takeout food on a particular Friday night, maneuvering an interaction with an acquaintance to get us out of a social situation that we would hate, or providing convincing reassurance about the exact thing we are worried about—all actions that make us feel like our loved ones “get” us. Misreading or being oblivious to the thoughts and feelings of one’s romantic partner seems like a recipe for a rocky relationship (although, interestingly, over time, accuracy appears to grow less important—Kilpatrick et al. 2002; Thomas et al. 1997)—or perhaps inaccuracy is better seen as an indication that the relationship has ceased to be a priority, which is no doubt accompanied by a number of other factors that foretell relationship dissolution. 

Similarly, whether or not caregivers are habitually “empathic” (in the compassionate sense), they need to understand and then attend to their charges’ needs. Skilled therapists need to correctly infer their clients’ thoughts and feelings and use this knowledge to validate the clients’ experiences and to suggest more adaptive mindsets and behaviors that can help ease mental distress. We expect the causal arrows to go both ways: dispositionally empathic people seek out caregiving opportunities, and caregiving settings call upon the people within them to behave in empathic ways. 

Thus, thought-feeling accuracy has been frequently studied in settings and relationships where it is highly likely that perceivers will already care about the targets—because targets are the perceivers’ romantic partners or because it is the perceivers’ literal job to care for the targets. There is also a third variable in these settings, which is both correlated with caring and has been shown to be related to thought-feeling accuracy: romantic partners and therapists *know* the targets (therapists know the targets—including quite personal details—through intake interviews and an ongoing therapeutic relationship). One of the earliest studies using the Ickes paradigm showed that perceivers who were close to targets were more accurate in making inferences about thoughts and feelings than strangers were (Stinson and Ickes 1992), and that furthermore, this advantage was mediated by the perceivers’ knowledge of what the target was talking about. When the target made references to “another place and time”, it provided more information to close others than strangers. (This may help explain why the close acquaintance advantage has not always been found when people are inferring the thoughts of close acquaintances when those close acquaintances are talking to other people—see Hancock and Ickes 1996; Thomas and Fletcher 2003.) Over time, people in a romantic or therapeutic relationship may also come to share motives (e.g., saving money for a house down payment or trying to reduce the risk of relapse)—or possibly just to assume that they share these things (see Thomas et al. 1997). Paradoxically, contexts that constrain caring and sharing to high levels may limit variance and thus make it harder to demonstrate significant correlations between accurate inference and prosocial outcomes. 

### 5.5. Reason 5: Prosocial May Be in the Eye of the Beholder 

While our fourth reason why thought-feeling accuracy is assumed to be “empathic” had to do with over-attention to certain contexts, our fifth proposed reason has to do with under-attention to certain outcomes. Studies of thought-feeling accuracy have rarely assessed outcomes experienced by targets—people who may be well-placed (indeed, arguably in the best position) to assess prosocial outcomes^3^. In the studies we discussed above that linked greater thought-feeling accuracy with prosocial outcomes, those outcomes were generally coded by a research team (e.g., supportive behavior in a couple) or evaluated by someone other than the target (e.g., successful outcomes in counseling). With a few exceptions, even in Ickes’ Dyadic Interaction paradigm, targets are generally not asked, “How well did the perceiver understand you?” And of course, in various contexts related to thought-feeling accuracy, targets could be asked not just about how well understood they felt but a host of other variables related to prosocial outcomes: how much they had been helped; whether they felt included; whether they were treated with compassion; positive changes in mood or well-being, etc. 

It is worth remembering that in an ongoing dyadic interaction, targets may have some control over the perceivers’ accuracy, including how useful the cues are that the target sends. If targets sense that the perceiver is not understanding them, they can say so (“No, no–it’s not that…”) and provide more explanation about what they are thinking and feeling. Alternatively, targets could be put off by the perceiver’s obliviousness, which may not only affect what the target further shares but also the overall tenor and outcome of the interaction. Targets’ perceptions of how helpful or understanding perceivers are may well be driven by variables other than accurate inference of discrete thoughts and feelings—for example, whether the perceiver tries to establish common ground or shared experience with the target (see, e.g., Hinnekens et al. 2020a). 

### 5.6. Reason 6: Confirmatory Instances Are Overrepresented in Memory

Sixth and finally, instances that fall into the present–present cell—i.e., the combination of the presence of thought-feeling accuracy and the presence of prosocial outcomes—may be easier to generate and more memorable than other combinations (as discussed in Hodges and Wise 2016). People tend to overestimate the co-occurrence of things that seem to belong together (i.e., illusory correlations; Nisbett and Ross 1980) and also assume that people with one positive characteristic will possess other positive characteristics (i.e., the halo effect; Feingold 1992). Among other things, thought-feeling accuracy and demonstrating warmth and nurturance are both associated with the female gender role (see Hodges and Wise 2016). On the flip side, people who are perceived as uncaring can be incorrectly believed to be inaccurate at inferring others’ thoughts and feelings as well (see Fernandez-Duque et al. 2010). 

Similarly, people may not be well attuned to register the combination where thought-feeling accuracy is present, but outcomes are neutral (or even negative) in terms of prosocial tendencies. There is not really much to notice when a person is accurate at inferring our thoughts and feelings but does not perform any actions that follow from these inferences. And in instances when someone is accurate and then behaves selfishly or unkindly towards us, it may be most important (and perhaps most adaptive) to pay attention to the harmful behavior—not whether or not that person was able to accurately infer our thoughts or feelings beforehand. 

## 6. When Thought-Feeling Accuracy Does Nothing … or Worse

After presenting six reasons why we think the link between thought-feeling accuracy and prosocial outcomes may be overestimated (likely by researchers and laypeople), we venture into the less explored territory where they are theoretically unrelated or even negatively correlated. As a first step in decoupling thought-feeling accuracy and prosociality, we note that such accuracy is not a requirement for being—or merely being perceived as—prosocial. People can be caring and provide competent help without necessarily having to resort to using subtle cues to accurately infer what an individual target is thinking or feeling. In many social interactions, targets and other observers may be entirely unaware of how accurate (or inaccurate) perceivers may be—so inaccuracy will often go undetected and thus will not necessarily lead to targets feeling misunderstood. As we noted earlier, inferring the contents of another person’s mind can require constructing something from cues that are not always directly accessible. These construction projects are too cognitively costly to engage in continuously, and furthermore, they are not our default method of communicating and coordinating with others—directly asking and telling is much more efficient (a point we will return to later). 

Unless perceivers verbally share inferences about what they believe the target is thinking and feeling or if perceivers act on their inferences in ways that reveal them, targets and others around them may be blissfully oblivious to rampant misassumptions by perceivers (Myers and Hodges 2009). What is not well known is how much “empathic” extra credit is earned for prosocial behaviors that appear to stem from correctly inferring and taking into account targets’ individualized thoughts and feelings. However, we do suspect that someone who reveals inaccuracy in inferring others’ thoughts and feelings may limit the degree to which their related actions will be seen as interpersonally sensitive, even if the intentions behind the actions were prosocial. In some extreme cases, the actions of an openly inaccurate perceiver may even be viewed antagonistically. The uncle who expects us to be excited about a gift of expensive opera tickets despite the fact that we have told him how we hate opera can, at best, be seen as generous—but probably will not be described as “empathic”. The co-worker who fails to realize our fear of big city driving may have meant well when giving us directions right through downtown as the most efficient route to a destination, but she likely will not be perceived as helpful. Even correctly inferring that someone is in distress but misreading the source of the distress may result in a considerable deduction in compassion points.

Our second step toward disassociating thought-feeling accuracy and prosociality presents an even greater challenge to the empathic prefix in the term “empathic accuracy”: Accurate inference in no way guarantees that the information gained will be used for prosocial ends. As our first illustration of this, consider all the professions that would seem to benefit from or require thought-feeling accuracy but for which prosocial goals are irrelevant to job success or even incompatible with it. For example, human resources officers and recruiters need to assess what job candidates are thinking and feeling during job interviews. The candidates’ records may provide objective performance evidence, but often, people are hired on the aspirational basis that they can and will learn the new job. Do they really feel comfortable using Python as a programming language? Are they excited about supervising a team of eight assistants? Will they be able to handle greeting walk-in clients while also keeping up with intake calls? Extracting the real picture from a job candidate’s answers requires thought-feeling accuracy on the part of the interviewer-as-perceiver, and the conclusions drawn may be entirely independent of the interviewer’s prosocial motives (for example, wanting to give the job to a member of an underrepresented minority) or may even conflict with prosocial motives (e.g., wanting to give the job to a candidate who desperately needs it—but knowing the person is seriously underqualified). 

Even in professions where helpful behaviors are expected (e.g., therapists), thought-feeling accuracy can direct people to act in ways that appear to be un-compassionate or that require suppressing compassionate responses to the distress that a target is perceived to be feeling. For example, in the medical arena, accurate inference of a patient’s craving for a painkiller or of a patient’s experience of certain symptoms may be the key to healing these patients or saving their lives, but it may also lead to denying them certain palliative care or to ordering highly invasive treatments in the process. And then there are the professions where accuracy is most decidedly not associated with prosocial aims. In competitive contexts like the military, law, business, and other negotiation settings, knowing what one’s competitor is thinking or feeling can be used to best them—whether that is leading an attack that will maximally demoralize them, discrediting their star witness, or stealing their marketing ideas. This kind of Machiavellian accuracy (see Hodges and Myers 2007) is definitely *un*-“empathic” and is found outside occupational transactions as well. For example, in dysfunctional family and romantic relationships (or even in moments of discord in functional relationships), the accurate inference of another person’s fears can be put to use in heightening their anxiety, or the accurate inference of positive anticipation can be used to create disappointment. 

The practice of gaslighting (American Psychological Association n.d.) is another example of thought-feeling accuracy being used for harm: The gaslighter must first have an accurate assessment of what the victims are thinking and feeling in order to make the victims doubt themselves. Other sinister users of not-so-“empathic” accuracy include con artists and even child sexual abuse offenders, whose ability to accurately infer victims’ thoughts and feelings may be part of grooming the victims (McAlinden 2006). More petty but still unkind behaviors may also involve exploitation of thought-feeling accuracy—e.g., purposefully grabbing the seat in a meeting where we inferred someone else was intending to sit. Using another person’s thoughts and feelings to personalize acts of aggression against them can greatly intensify the hostility. 

Although we can easily generate anecdotes of thought-feeling accuracy serving antisocial goals, empirical examples are currently fairly limited. This could mean that dark uses of this form of accuracy are rare or that they are hard to study because of social desirability concerns—or perhaps a combination of the two. Creating opportunities for dark behaviors to occur also has its ethical limits. Probing the circumstances in which thought-feeling accuracy is used for nefarious purposes constitutes an important and intriguing direction for future research. 

We suspect that although thought-feeling accuracy can be used for purposes that hurt others, more often it is used simply for self-serving purposes that are neutral to others or, at any rate, are not considered immoral. People use accuracy to understand things they wonder about and to figure out how to coordinate with other people to get what they want. This would suggest that (independently of any relationship to prosociality), a perceiver’s thought-feeling accuracy generally benefits them—and there are some empirical studies that demonstrate positive outcomes for people who are more accurate in this way (e.g., Blanke et al. 2016; Gleason et al. 2009; Lorimer and Jowett 2009b). Thought-feeling accuracy may deliver benefits, particularly in the realm of *metaperceptions*. Systematic biases have been identified in people’s perceptions of others’ thoughts and feelings that exaggerate how much others have critical thoughts about them (e.g., perceiving them as socially inept or badly dressed). Broadly in line with research by Hinnekens et al. (2020b) that links specific mind-reading errors to lower overall accuracy, being more accurate may protect perceivers against these painful negative perceived evaluations.

The benefits of accuracy for perceivers may be even more significant in the context of intergroup interactions between members of advantaged majority and disadvantaged minority groups. Vorauer and colleagues’ work (e.g., Vorauer and Kumhyr 2001; Vorauer et al. 1998, 2009) vividly illustrates the possible pitfalls of exaggerated negative metaperceptions—or other metaperceptual errors (Vorauer and Sakamoto 2006)—in intergroup exchanges (see also Bergsieker et al. 2010; Kteily et al. 2016). Majority group perceivers may erroneously infer that minority group targets perceive them as prejudiced, and such inferences can lead to more negative self-evaluations as well as dampen enthusiasm for pursuing already fragile intergroup relations.

However, we are somewhat surprised there are not more studies demonstrating the rewards of accurately inferring others’ thoughts and feelings—both generally and in specific contexts. Thinking of accuracy more as a useful tool to deploy when needed and less as a trait or talent helps explain this mystery: A can opener is really handy if one needs to open a can of beans, but can opener operation does not consume our attention for most of the day, and the advantages of owning a can opener do not generalize across broad swathes of our lives. 

Greater thought-feeling accuracy can also have some downsides for the perceiver (for an excellent review of the potential downsides of accuracy in reading affective cues—some of which are shared with thought-feeling accuracy—see Schlegel 2020). Just as is the case for the benefits of thought-feeling accuracy, these downsides are likely specific to particular instances and do not confer some generalized disadvantage. Chief among the negative consequences is finding out someone is thinking or feeling something the perceiver does not want to know. This unwanted knowledge is often about the self: that they dislike you, they think those pants make you look fat, or they found your pun bad. 

Another compelling example of the dangers of accurate inference can be found in Simpson et al.’s (2003) work, which demonstrates that accurately inferring that one’s partner finds another person attractive may reduce feelings of closeness in the relationship. To make things worse, partners who are insecure about the relationship (because, for example, they have an anxious attachment style) may be particularly vigilant and accurate when it comes to reading their partner’s thoughts about other people’s attractiveness (Simpson et al. 1999).

Not all unwanted outcomes resulting from accurate inferences reflect badly on aspects of the perceiver. The target could also be experiencing negative affect (generally not pleasant for perceivers to infer except maybe in cases of schadenfreude) or having thoughts about a negative aspect of the world (e.g., the target is not surprised a peer falsified their data or the target believes there really is no chance of escaping a recession this next year). Jobs that involve a lot of inference of unpleasant thoughts seem ripe for leading to burnout: for example, therapists working with suicidal clients (whose thoughts are about self-destruction) or teachers working with school administrators who have no resources (whose thoughts about budget woes have consequences for the teachers). Encountering thoughts that are contrary to one’s own may be existentially challenging (Hodges et al. 2018), and if one spends time inferring multiple people’s thoughts, the cacophony of viewpoints may also be disturbing. 

There are also the simple opportunity costs of devoting time and effort to being accurate. These costs are not limited to times when perceivers are accurate; they are present whenever a perceiver directs attention away from other topics in order to try to infer a target’s thoughts and feelings. Use of the Ickes paradigm (especially the dyadic interaction version) yields better than chance but not especially impressive levels of thought-feeling accuracy (see Hodges et al. 2015), so attention directed toward trying to read someone’s mind may not be worth the effort. And although we know of no empirical research on this topic, we suspect that there could be interpersonal costs to being perceived as someone who expends extensive effort to “mindread”—visible efforts might be viewed as odd, indicating poor social skills, or creepily threatening. 

## 7. Concluding Thoughts

This last point brings us to a final idea that we think is an intriguing future direction for thought-feeling accuracy research and is also relevant to its connection to prosocial behavior. The most effective and efficient way for humans to know each other’s minds is, fortunately, *not* via inference. Humans have developed a highly advanced and direct communication system for getting perspectives—in the form of words and language. No inference is required if one simply asks the target what they are thinking and feeling. Consistent with this idea, Eyal et al. (2018) encourage the use of perspective *getting* through conversation over perspective *taking* through inference as a more effective route to understanding other people’s minds. 

Humans can also quickly communicate simple but critical emotion-relevant messages nonverbally that may be processed with minimal or no inference required (messages along the lines of “I’m going to attack you”; “Something else is going to attack us both”; “The baby is in pain”; and “You’re pretty cute”), something we share with other species. If we really needed to know what was in people’s minds that they were not telling us with their words or emotion displays, maybe we would have evolved thought receptors. Instead, it may have been more adaptive to have the option of thinking about some things privately without them being accessible to others. 

When we think about the instances when we most desire accuracy, it is often when targets are not telling us what is on their mind. Maybe this is because they are not entirely sure of these thoughts and feelings themselves. Or maybe they are embarrassed about what they are thinking or feeling, they do not want to upset us or think their thoughts and feelings will cause an argument, or they are lying to us or want to use privileged information to their own advantage. In these cases, just how prosocial is it for perceivers to pursue thought-feeling accuracy? A few studies have already indirectly explored questions about how much targets want their thoughts to be known or how much perceivers really want to know them (e.g., Lawless DesJardins and Hodges (2015), who studied interactions between strangers who might be lying, and Simpson et al. (1995), who studied romantic couples who seemed to avoid accurately seeing that their partner might be attracted to someone to protect the relationship). We think targets’ desire not to share some thoughts and feelings and perceivers’ desire to probe them anyway may be some of the most interesting variables to study when it comes to accuracy in inferring others’ thoughts and feelings. 

For now, though, studying thought-feeling accuracy in contexts where targets may not want their thoughts and feelings to be known remains understudied. Almost certainly, part of the reason for this is that this research would be challenging to conduct. Already, the Ickes paradigm is complex to run and requires small armies of research assistants to collect data and code inferences; augmenting potentially socially undesirable themes in thoughts and inferences would further complicate matters. However, we suspect a further impediment to studying the inference of thoughts that targets are reluctant to share is the remarkably persistent assumption the current quartet of authors has encountered in our research lives: that thought-feeling accuracy is something “nice” and prosocial that “empathic” people do. Challenging that assumption—as we hope we have accomplished here—may open ways to contemplate and appreciate new aspects of human social cognition and what it means to be emotionally intelligent. 

## Data Availability

There were no datasets associated with the writing of this manuscript.

## Notes

1 The RMET is often referred to as a measure of theory of mind–see review by Kittel et al. (2022)–but is better described as a measure of emotional decoding.

2 We want to be absolutely clear that we are solely “playing” with theoretical models and are not pretending to present evidence that could speak to the causal direction–if any–between these two constructs.

3 Targets also have a privileged and unique perspective on perceiver accuracy, as they alone have provided the criterion for accuracy. When targets rate the accuracy of perceivers’ inferences, they give higher accuracy ratings than “objective” coders and are not as highly correlated with objective coders as other objective coders are with each other (Hodges et al. 2015).
